# Supplementary material for: Effects of Progressive Resistance Training After Hip Fracture: A Systematic Review
Source: J Funct Morphol Kinesiol. 2025 Feb 2;10(1):54. doi: 10.3390/jfmk10010054 (PMC11843970; doi:10.3390/jfmk10010054)
Supplement: Supplementary file 1 [file jfmk-10-00054-s001.zip › jfmk-3354838-supplementary.pdf]

Supplementary file 1. Full search strategy

DateBase	Search strategy
Pubmed	(hip[Title]) AND (resistance[Title])
Pubmed	(hip[Title]) AND (strength[Title])
Pubmed	(hip[Title]) AND (strengthening[Title])
Pubmed	(hip[Title]) AND (concentric[Title])
Pubmed	(hip[Title]) AND (eccentric[Title])
Pubmed	(hip[Title]) AND (endurance[Title])
Pubmed	(hip[Title]) AND (elastic tube[Title])
Pubmed	(hip[Title]) AND (pulleys[Title])
Cochrane	(hip):ti AND (resistance):ti
Cochrane	(hip):ti AND (strength):ti
Cochrane	(hip):ti AND (strengthening):ti
Cochrane	(hip):ti AND (concentric):ti
Cochrane	(hip):ti AND (eccentric):ti
Cochrane	(hip):ti AND (endurance):ti
Cochrane	(hip):ti AND (elastic tube):ti
Cochrane	(hip):ti AND (pulleys):ti
Ebesco	TI hip AND TI resistance
Ebesco	TI hip AND TI strength
Ebesco	TI hip AND TI strengthening
Ebesco	TI hip AND TI concentric
Ebesco	TI hip AND TI eccentric
Ebesco	TI hip AND TI endurance
Ebesco	TI hip AND TI elastic tube
Ebesco	TI hip AND TI pulleys
